# Validation of the inventory: lockdown and its impact on the university Community

**DOI:** 10.1186/s12889-023-17562-y

**Published:** 2024-03-13

**Authors:** María Yolanda González-Alonso, Andrea Liliana Ortiz-González, María Camino Escolar-Llamazares, Diego Serrano-Gómez, Isabel Serrano-Pintado, María Ángeles Martínez-Martín

**Affiliations:** 1 University of Burgos, Burgos, Spain; 2 Sergio Arboleda University, Bogotá, Colombia; 3 University of Salamanca, Salamanca, Spain

**Keywords:** University, Validity, Reliability, COVID-19, Mental health

## Abstract

**Background:**

Significant consequences of COVID-19 within academic/professional life are, at the psychological level, related to worry, tension, stress; coping strategies and lifestyle changes. This study describes the process of design and validation of an inventory (QPIC), which aims to assess the psychological impact that a situation of confinement can produce among university students and teachers.

**Methods:**

Design of the instrument and psychometric tests. A sample of 862 students and 229 professors affiliated to Spanish and Colombian universities was used. Data were collected in April 2020 with the request of the favourable Bioethics Committee IR/2020.

**Results:**

Six experts carried out the content validation. A confirmatory factor analysis of the theoretical dimensions proposed for the scales was performed and the internal consistency of each of the three initial scales was confirmed (0.866, 0.813 and 0.834).

**Conclusion:**

A rigorous and reliable instrument is achieved, consisting of two final scales: (a) Worry, tension and stress scale (b) Coping scale, which helps to measure individual psychological effects in housebound situations. It is an instrument designed, constructed ad hoc to assess the impact of confinement and subjected to validation. The factor structure and reliability of the instrument are examined and good psychometric properties are obtained. The application of this inventory will make it possible to assess the impact on people’s mental health during a period of confinement.

**Supplementary Information:**

The online version contains supplementary material available at 10.1186/s12889-023-17562-y.

## Introduction

At present an exceptional situation is being lived through, known as the “first global pandemic in history” called COVID-19. On 30 January 2020, the OMS communicated this illness as a public health emergency of international scope [1].

COVID is generating immense impact upon society, health, the environment and in other areas at a global level [2]. As a consequence of it all, governments have prepared strategies for coping with the pandemic, among which the strictest has been confinement at home or lockdown. “Confinement is an intervention that is applied at a community level when the aforementioned measures, although implemented, have been insufficient to contain the contagious spread of an illness” [3]. In Spain, a national state of alarm was declared on 14 March, the lockdown began on 14 March and ended on 21 June. In Colombia, it started on 25 March and ended on 31 August. Lockdown entailed the progressive closure of schools and universities, causing anxiety, depression and stress [4].

The pandemic has marked the life of university students and teachers alike. The institutions had to adapt themselves to the circumstances, in order to continue training students, through remote classes, using exclusively on-line teaching during the confinement through a virtual platform [5]. This fact has shaken the educational and the socio-emotional development of both students and teachers who had progressively to discover the full scope of a 100% distance-learning environment.

This obligatory adaptation provoked an emotional overload for the university community, generating symptoms of stress, anxiety and depression, due to the lack of quality technological resources and the scarcity of training in on-line technologies. Moreover, loss of short-term student employment threatened their economic stability. Added to all of the above were concerns and fears over infection and transmission of COVID-19 to family members and the effects of isolation [6]. The uncertainty associated with this illness, the effect of social distancing, isolation and confinement have negatively impacted on the mental health of the university community at a global level [7].

The psychological impact of COVID-19 upon the university community has been an object of study for different researchers who have evaluated COVID-19-related anxiety, depression and stress within the university community [8–10].

Ascertaining the impact of confinement on the mental health of university teachers and students requires reliable instruments, validated with consistency and rigour. A search will be carried out in the databases, Web of Sciences and Scopus using “COVID-19”, “confinement”, “assessment”, “stress” and “coping” as keywords. There are few investigations on the validation of questionnaires and scales that measure concern, tension, stress; the coping situation and changes to the academic/professional life of university teachers and students linked to COVID − 19. Some examples might be the study of Vera-Ponce et al. (2020) [10] referring to a scale intended to measure lifestyle change among university students from Lima, and the one by Martínez-Lorca et al. (2020) [11] who developed the scale Fear of COVID-19 (FCV-19 S) with Spanish university students. Focus groups were also held with both Spanish and Colombian teachers and students to share ideas and experiences on the situation of housebound.

Therefore, this work has as its main objective, the construction and validation of an instrument that measures psychological impact on three scales that evaluate concern, tension and stress; Coping strategies; and Changes in both the academic and the professional life of university students and teachers in times of COVID-19 during the lockdown period. Evidence of the content, the construct validity and the internal consistency of the domains that constitute the instrument are analysed. The concurrent validity of the initial scales was also investigated. The validation improves our knowledge of the university community and its behavior during periods of confinement.

## Methods

A descriptive and transversal study was conducted over two phases. The first consisted of the design and preparation of an instrument that serves to evaluate the impact of COVID-19 on the university community, and the second was centred on content validation, and on the reproduction, effectiveness and construct validity of the questionnaire design.

### Participants

The instrument was applied to 862 students studying for different university qualifications and to 229 teachers affiliated to Spanish (Salamanca and Burgos) and Colombian universities (Sergio Arboleda and Metropolitana), which complied with the established criteria for inclusion (being over age, forming part of the selected universities and signing written informed consent). The minimum required size was estimated at 200 participants, in accordance with Hair (2009) [12] who recommended the use of at least 5 subjects per parameter. The sample was considered representative when it contributed sufficient data to obtain a good estimate of the parameters to be measured [13].

Looking at the valid responses, the sample of students comprised 616 men (70.2%) and 246 women (28.1%), all students, whose average age was 23.27 years old. 66.5% of the population was single and came from different places in both Spain and Colombia.

As for the teachers, the sample was formed of 128 men (55.2%) and 101 women (43.5%) whose average age was 45.19 years old. Almost half, 47.4%, were married.

### Procedure

The construction of the questionnaire that seeks to measure the psychological impact of confinement on university students and teachers began with a systematic review, with the aim of describing the domains. In other words, the concepts, attributes and behaviours under study are defined, which in this case were (1) Concern, tension and stress in the face of the situation generated by COVID-19; (2) Coping strategies towards the COVID-19 situation; and (3) changes in academic/professional life in that scenario.

Concern, tension, and stress arising from the COVID-19 situation. Concern, tension and stress are present in vital life experiences, subjective experiences, and emotional and physiological responses and can be explained from environmental, psychological and biomedical perspectives [14]. Latin-American and Spanish researchers coincided over highlighting increased stress among teachers during the months of confinement and those that followed and showed levels of medium-high stress associated with psycho-social factors originating due to isolation, fear, uncertainty and workload [15]. Changes to routine and new demands on the teaching staff have implied the need to adapt the teaching method, generating stress and high anxiety levels that affect their emotional wellbeing and their mental health [16]. The area of Concern will be measured with this scale, an attempt to evaluate the concern and the perception of stress that university students and teachers felt during the situation of confinement due to COVID-19.

The Coping Situation resulting from COVID-19. Coping refers to the behavioural and cognitive efforts that are employed to manage both external and internal demands that are perceived as exhaustive or excessive on the basis of whether the available resources are effective or otherwise [17]. It entails the strategies that a person uses to resolve situations of stress after a cognitive evaluation [18], seeking alleviation, compensation or equilibrium [19]. There are several different instruments that assess coping strategies [20–24]. The scale, the object of validation, establishes the domains Adaptation and Emotional expression and social support as coping strategies for university teachers and students to live with the present crisis situation.

Changes to academic/teaching life during the COVID-19 situation. Lifestyles and actions that are associated with health are determined by various sorts of factors. There is a connection between the behaviour of a person, the community where that person is living and the person’s health. The scale of changes to the academic/working life of teachers and students is centred on identifying some changes that emerged during confinement because of the pandemic, which can imply health risks and when early detection can prevent psychological problems. This scale evaluates the domains Physical consequence and Planning.

Having defined the domains, the scales were designed, on the basis of a battery of questions prepared after considering the scientific literature. A provisional list of 40 easy to answer items on a 1–6-point Likert-type scale was distributed throughout the five aforementioned domains (Concern, Adaptation, Emotional Expression and Social Support, Physical Consequences and Planning). Initially, the scales began with a number of items that progressed towards a definitive number. Concern, tension and stress: 8 items appeared in the teacher’s version and 7 in the students’ version. Coping Strategies had 12 items, and Changes to academic/teaching life: 17 and 20 items, respectively, in the student and in the teacher’s questionnaire.

Once the domains had been identified, the Spanish and Colombian research teams proposed guidelines (representativeness, relevance, diversity, clarity, simplicity and comprehensibility) for the development of items for each dimension. In the initial stages of item bank construction, the researchers in each country consulted teachers and students. The results were recorded on structured forms and completed in each country. This input contributed to a first item bank in Spanish, which went through a process of item reduction and selection. First, redundant, inappropriate or difficult to understand items were eliminated and some missing items were included. The items will be revised until an acceptable and culturally understandable version is obtained for both Spaniards and Colombians. The researchers then meet and agree on the final version of the questionnaire. At the end of the process, the content will be evaluated in a pre-test. The researchers administer the questionnaire to 8 teachers and 14 Spanish and Colombian students. Incidents and difficulties are collected and used to improve the wording, detect errors, eliminate problematic items and arrive at the final version.

### Ethical considerations

Throughout the process, compliance with legal and ethical aspects and the rights of participants were assured: Declaration of Helsinki, Organic Law 3/2018, of 5 December, on the Protection of Personal Data and Guaranteeing Digital Rights (Spain) and Law 10/90 of September 6 de 2006 (Colombia).

The Bioethics Committee of the University of Burgos (Spain) approved the study that had previously obtained the informed consent of the participants.

### Validation of experts

Having written the items that constitute the instruments, content validation was initiated through the critical judgement of experts who guaranteed that the items fitted the construct that is to be measured [25]. This expert opinion implies a valuation of the items that constitute the instrument that is under study. Criteria selection was tasked to a group of six professionals from clinical psychology, with trajectories characterized by long experience in teaching and research in the field of mental health, specifically in anxiety and mood disorders.

An individualized email was sent with the questionnaire attached to the message to which they had to respond without consulting between each other. The questionnaire included the different items of the instrument to be valued in which they had to rate on a scale of 1 to 4 the compliance of each item with criteria of clarity (if the items were understood and if proper syntax and semantics were used); coherence (if they had a logical relation with the domain that was measured); and relevance (each item was important and had to be included). At the end of each domain, the evaluation of the criterion of sufficiency was included alongside a space for observations [26].

### Statistical analysis

A descriptive analysis was performed to verify the general characteristics of the participants. The external consistency of the panel of experts’ validation was measured by using analysis per item through the average according to the criteria of the experts and homogeneity and the correlations between each item-element were likewise evaluated. The degree of agreement of the judges was analyzed through the coefficient of external concordance, Kendall’s W, which yields the ranges of variance, in order to identify the degree of agreement among experts.

The reliability of the construct was calculated with Cronbach’s α, considering a value of over 0.8 as an indicator consistency. The Kaiser Meyer Olkin (KMO) and the sphericity test of Bartlett were used to evaluate the previous conditions and to verify instrument validity, as well as to analyze the matrix and whether any item-total correlations existed, in order to achieve construct validity. Exploratory Factor Analysis (EFA) was used, in order to uncover underlying structures and their construct validity. Possible factors were identified through a Varimax rotation principal components analysis.

## Results

### Expert validation

The validation of the instrument was based on the observations that the experts assigned to the scales and to the items, following the criteria: Clarity, Coherence and Relevance. Kendall’s W statistical test was applied to determine the degree of agreement between the experts. Adequate criteria were identified for each item of relevance for the three scales, as may be seen below, in Table 1.

**Table 1:** Analysis of evaluations by criterion

	Clarity	Coherence	Relevance
E1	2.86	2.86	3.40
E2	3.63	3.63	3.61
E3	4.44	4.44	4.24
E4	2.61	2.61	2.58
E5	3.54	3.54	3.46
E6	3.93	3.93	3.71
Kendall’s W	0.303	0.205	0.164
P	< 0.005	< 0.005	< 0.005

Kendall’s W contributes a measure of content validity according to the concordance or agreement between the experts.

In Table 2, a weak concordance may be seen between Scale 1 and Scale 2 and the experts suggested that the items could be sub-divided or grouped into other dimensions. With regard to the Planning dimension, the agreement between the experts was still closer. The qualitative contribution from the experts was considered fundamental.

**Table 2:** Analysis of evaluations by scale

Scales	Domains	Kendall’s W	P
Scale 1: Concern, tension and stress	Concern	0.090	0.126
Scale 2: Coping with the COVID-19 situation	Adaptation	0.062	0.347
	Emotional expression and social support	0.052	0.474
Scale 3: Changes in academic/teaching life	Physical consequences	0.074	0.249
	Planning	0.155	0.001

An analysis was completed of the scores for each of the scales from the experts. On the first scale “Concern, tension and stress provoked by the current COVID-19 situation*”*, the experts showed great agreement for each item, all of them properly measuring the personal, family and social, economic and working situations. One of the experts added that the definition of tension should be explained in a general way, considering that the variables had been defined in general and non-specific ways. The section on the definition of the domains of each scale, drawn from consulting the bibliography, was included and may be seen in the working method.

On the second scale “Coping with the situation created by COVID-19”, its items are divided between the domains Adaptation and Emotional expression and social support. The experts were in agreement over the analysis of the items, in both domains, given that, in their opinion, the participants were aware of their coping strategies and the different ways of obtaining social support.

Finally, the third scale was evaluated “Changes to the academic/teaching life of university teachers and students following the situation created by COVID-19”, comprising the domains Physical consequences and Planning. The experts agreed with the items and their development, because they evaluated the typical physical consequences of a stressful situation: tiredness, overload, hyperventilation, poor quality of sleep. They also described feelings, difficulties and perceptions (thoughts) that teachers and students might have when confronting this COVID-19 situation and wishing to perform their work well.

With regard to clarity, coherence and relevance, the experts, agreed in so far as some items had not been understood, had little or no relation with the domain under evaluation, or the items were considered non-essential and they were given a low score. These results reflected that, in the opinion of the experts, the low scores in these items could be due to comprehension difficulties, so it was suggested that those items be removed.

### Analysis of homogeneity

With regard to the homogeneity and discriminatory capacity of the items, most of the correlations with the items were adequate and only a few slightly moved away from the criterion, which led to the idea that a better fit might be achieved without them.

As may be appreciated from Table 3, items 2 and 4 could be removed from the scale of Concern, tension and stress; items 1, 2, 8 and 9 could be removed from the Scale of Coping Strategies towards the COVID-19 situation; items 1, 2, 5, 11 and 16 could be reviewed in Scale 3 Changes facing the COVID-19 situation; and items 6, 7, 14 and 20 in the domain of Planning. These items will be reviewed, taking into consideration any coincidences with the expert evaluation that can be shown.

Where negative values emerged making no contribution to the optimal measuring scales, the possibility of their removal was studied (Table 3).

**Table 3:** Analysis of reliability by item

Items	Measure of scale when the element has been removed	Variance of scale when the element has been removed	Total correlation of corrected elements	Cronbach’s α when the element has been removed
P1	129.0000	228.000	0.000	0.879
P2	129.3333	214.333	0.789	0.871
P3	129.3333	214.333	0.789	0.871
P4	129.0000	228.000	0.000	0.879
P5	129.6667	230.333	− 0.152	0.882
P6	128.3333	197.333	0.904	0.863
P7	129.0000	211.000	0.551	0.872
P8	129.0000	211.000	0.551	0.872
Af1	129.0000	183.000	0.896	0.860
Af2	129.0000	183.000	0.896	0.860
Af3	128.6667	196.333	0.685	0.868
Af4	128.3333	197.333	0.904	0.863
Af5	128.3333	197.333	0.904	0.863
Af6	128.6667	212.333	0.911	0.870
Af7	128.6667	212.333	0.911	0.870
Af8	128.6667	212.333	0.911	0.870
Af9_R	128.6667	212.333	0.911	0.870
Af10	128.3333	197.333	0.904	0.863
Af11	128.6667	212.333	0.911	0.870
Af12	129.6667	212.333	0.911	0.870
Cd1	129.6667	212.333	0.911	0.870
Cd2	129.3333	214.333	0.789	0.871
Cd3	128.6667	230.333	− 0.152	0.882
Cd4	128.3333	214.333	0.789	0.871
Cd5	129.6667	242.333	− 0.816	0.889
Cd6	128.3333	214.333	0.789	0.871
Cd7	128.6667	230.333	− 0.152	0.882
Cd8	129.0000	211.000	0.551	0.872
Cd9	128.6667	201.333	0.773	0.867
Cd10	128.3333	204.333	0.489	0.874
Cd11	129.6667	230.333	− 0.152	0.882
Cd12	128.3333	233.333	− 0.189	0.888
Cd14	128.4445	233.313	− 0.180	0.889
Cd13	128.6667	212.333	0.911	0.870
Cd15	128.0000	217.000	0.339	0.877
Cd16	127.3333	244.333	− 0.923	0.891
Cd17	128.0000	279.000	− 0.933	0.917
Cd18	127.6667	242.333	− 0.816	0.889
Cd19	127.3333	244.333	− 0.923	0.891
Cd20	130.0000	228.000	0.000	0.879

### Analysis of internal validity

The analysis of internal validity was performed using the Kaiser-Meyer-Olkin (KMO) index. Bartlett’s sphericity test was significant on the three scales, which underlined the need to perform a factor analysis (Table 4).

**Table 4:** KMO test and Bartlett’s test of sphericity

	KMO	Bartlett’s Sphericity Test		
		Approx. Chi squared	gl	P
Scale 1	0.863	202.640	28	< 0.001
Scale 2	0.479	182.426	66	< 0.001
Scale 3	0.579	416.640	190	< 0.001

### Analysis of internal consistency-reliability

An internal consistency analysis was performed to evaluate the reliability of the scales. A Cronbach’s α of 0.866 was obtained on the Scale for Concern, tension and stress, it was 0.813 on the scale of Coping strategies towards the COVID-19 situation and 0.834 on the scale of Changes to the situation experienced because of COVID-19.

### Analysis of the test-retest

The temporal consistency and reproducibility of the questionnaire was evaluated on two occasions with the same people, between two and four weeks apart. 14 university students and 8 Spanish and Colombian teachers were randomly selected. The results obtained were statistically very significant and showed a satisfactory internal consistency with a Cronbach’s alpha for students: test = 0.870, retest = 0.847 and set = 0.911; and for teachers: test = 0.659, retest = 0.815 and set = 0.964. To determine the temporal stability of the questionnaire, a significant Pearson correlation coefficient (p < 0.001) was obtained between test and retest, indicating that there is temporal stability.

### Exploratory factor analysis

The items were grouped into two large principal components for the Factor analysis: component 1 integrated by 21 items and component 2 by 20 (Table 5). It was considered that if the relevance of the items under 0.5 and negative numbers to the scale could not be shown, then they could be removed.

**Table 5:** Distribution of components

Item	Component 1	Component 2
P2	0.872	
P3	0.818	
P1	0.798	
P4	0.756	0.333
P5	0.705	
P8	0.686	
P7	0.608	
P6	0.564	
Af1	0.860	
Af4	0.870	
Af5	0.770	
Af6	0.689	
Af12	0.381	
Af8		0.905
Af3		0.732
Af7	0.342	0.614
Af11	0.433	0.609
Af10		0.557
Af9_R	0.517	− 0.523
Af2	0.427	0.507
C9	0.654	
C10	0.531	0.103
C15	0.521	
C6	0.518	
C21	0.446	
C3	0.411	0.148
C20	0.382	0.113
C4	0.346	0.523
C12	0.253	
C19	0.147	
C2		0.660
C8	− 0.172	0.657
C1		0.601
C7		0.400
C5		0.377
C18	− 0.120	0.328
C14	− 0.130	0.327
C13	− 0.212	0.307
C11		0.273
C17		0.253
C16		0.240

The questionnaire Psychological Impact of COVID-19 on university teachers and students (QPIC) comprises 21 items organized into two dimensions, the Scale of Concern, tension and stress with 15 items and the Scale of Coping Strategies formed of 6 items. This questionnaire generates scores for each dimension. An explanation of how it is scored is included in the results section of the manuscript. Each response is scored on a graded Likert scale from 1 to 6 (1 = lowest impact/ coping and 6 = highest impact/ coping). On the Worry, Tension and Stress scale, a score between 15 and 40 points is considered low impact, medium impact between 41 and 65 points and high impact between 66 and 90 points. The cut-off point for a high score is 66 (inclusive). On the coping strategies scale, low coping is defined as a score between 6 and 15, medium coping as a score between 16 and 26, and high coping as a score between 27 and 36. The cut-off point is 27.

## Discussion

COVID-19 has produced a situation that implies a readjustment of working methods within the university community. The teachers were expected to promote new teaching modalities, in order for teaching activity to continue. The most widely used option was online teaching in real time, in such a way that the established programme could be followed, but not face-2-face (35), which required the adaptation of the students to this teaching modality. These changes at an academic level, linked to the specific socio-sanitary circumstances of the pandemic, have provoked a significant psychological impact within the university community as well as in the rest of the population [27], which has to be known, to be able to establish preventive strategies. It is therefore fundamental to rely on validated instruments that make it possible to measure both student and teachers’ perceptions of the effects caused by COVID-19.

To arrive at the objective of the present study, the validation of a tool with which to measure the variables concern, tension, stress; coping strategies and changes to academic/professional life have made it necessary to follow established procedure for such ends. In accordance with Williams & Webb (1994) [28] and Powell (2003) [29], who pointed out that there is no defined number that decides how many experts are necessary, the evaluations of six experts were collected. One way to gauge the reliability of an instrument consists in taking the measurement of an individual at two different times. The Test-retest analysis was chosen, after an interval of seven days, following the proposal of Hulley (1993), who suggested that the best way to obtain better results was to apply the second questionnaire within a period that fluctuated between seven days and two months.

The absence of validated instruments that measure Concern, tension and stress; Coping strategies and Changes to academic/professional life during confinement among students and teachers, prevented any comparison of the information with other instruments. A work that presented one particular approach was centred on the validation of a scale directed at knowing the Changes in lifestyles during confinement among university students from Lima [10]. In this case, the questionnaire grouped 4 areas, dimensions and domains organized to measure the construct changes in lifestyle during strict lockdown: consumption of food, physical activity, consumption of harmful habits (alcohol and cigarettes) and the use of channels of communication, which is outside the scope of the present article that is to measure changes centred on academic life.

Another approximation was shown in the validation of the scale Fear of COVID-19 (FCV-19 S) among Spanish university students, the work of Martínez-Lorca et al. (2020) [11], based on the Scale Fear of COVID-19 (FCV-19 S) by Ahorsu et al. (2020) [30], which is used to evaluate the fear of COVID-19 among Spanish students. Among the seven items that it comprises, one makes reference to responses to fear and anxiety (acceleration of the heart, sweating, sleep disorders, fear of death, infection, etc.), items that are related with the domain Physiological Changes on the scale “Changes to academic/professional” life of the present study, which points to the relevance of physical changes on the measure of the anxiety responses.

The collection and interpretation of the results, sheds light on certain limitations. The university students and teachers, although they coincided with the characteristics of the target population, formed a convenience sample, for which reason the results cannot be extrapolated to the general population. It is therefore advisable to perform future studies that could use more representative samples at both a national and an international level. Most of the students and teachers were men, for which reason the incorporation of the gender perspective in future works is proposed and the analysis of any significant differences. It would also be useful to perform a qualitative study with respect to social representation and cultural differences between Spanish and Colombian students and teachers in relation to the variables under study. It would be interesting to analyze the correlations between the scales that were prepared and in the State-Trait Anxiety Inventory (STAI) and the Beck Depression Inventory (BDI) from Beck et al. 1979 [31], with the purpose of reaffirming the validity of the instrument.

The presence of COVID-19 in our lives has implied an important impact in all areas (sanitary, educational, social, economic…). Among the multiple consequences associated with it, the psychological effect is the point to highlight, centring attention on concerns, fears and anxiety generated in the group of university teachers and students, the coping strategies in use and the changes it has meant for academic life. Reliable instruments are required that show consistency and rigour, to become aware of the impact of confinement on university teachers and students. Up against the inexistence of tools that fulfil this objective, the validation has been completed of the two following scales: Concern over the COVID-19 situation and Coping strategies towards the COVID-19 situation.

The instrument has shown high reliability and validity, with solid psychometric properties, which, polishing the limitations that were found, has converted it into an optimal tool for measuring the domains (concern, adaptation, planning, support and consequences) among university students and teachers. It might be of immense help to achieve greater knowledge of the emotional state of the university community in times of crisis with a view to establishing preventive programmes and interventions that identify signs and risk groups on which to act, minimizing the adverse consequences.

### Electronic supplementary material

Below is the link to the electronic supplementary material.

Supplementary Material 1

## Data Availability

The datasets used and/or analysed during the current study available from the corresponding author on reasonable request.
