# Supplementary material for: Validation of the inventory: lockdown and its impact on the university Community
Source: BMC Public Health. 2024 Mar 13;24:790. doi: 10.1186/s12889-023-17562-y (PMC10938755; doi:10.1186/s12889-023-17562-y)
Supplement: Supplementary file 1 — Supplementary Material 1 [file 12889_2023_17562_MOESM1_ESM.docx]

Ref: Submission ID 74885a30-c517-4a86-b442-429e74f44a43

Re: "Validation of the Inventory: Lockdown and its impact on the University Community"

**APPENDIX I**. Cuestionario Impacto psicológico de la COVID-19 en estudiantes y profesores universitarios durante el confinamiento (QPIC).

**ESCALA DE PREOCUPACIÓN, TENSIÓN Y ESTRÉS**

| *Preocupación/ Adaptación/Planificación* |
| --- |
| P1. Nivel de preocupación, tensión y estrés que siento frente al confinamiento. |
| P2. La situación de confinamiento me genera preocupación, tensión y estrés familiar. |
| P3. Siento que esta situación me genera preocupación, tensión y estrés en mi vida social y afectiva. |
| P4. Pensar que esta situación pueda alargarse me causa preocupación, tensión y estrés. |
| P5. La situación de crisis debida al COVID-19, me produce preocupación, tensión y estrés por el miedo al contagio. |
| P6. La situación de crisis debida al COVID-19, me produce preocupación, tensión y estrés por el miedo a las consecuencias socioeconómicas asociadas. |
| P7. La situación de confinamiento me genera preocupación, tensión y estrés que influye en mi capacidad de concentración, disminuyendo mi rendimiento. |
| P8. La situación de confinamiento me aumentó la preocupación, tensión y estrés en mis labores como profesor. |
| Af4. Considero que tengo recursos para afrontar momentos de crisis. |
| Af5. En esta situación de crisis puedo controlar fácilmente mis reacciones emocionales de alegría, tristeza, angustia y rabia. |
| Af9. En los días de confinamiento noté que cambiaba mi humor y me enfadaba sin motivo. |
| C6. Planifico de forma óptima el trabajo semanal |
| C9. Siento que, incluso en estas circunstancias, puedo crear con facilidad un clima agradable con mis compañeros de estudios/alumnos. |
| C10. El feedback que recibo de los profesores/ mis estudiantes me hacen sentir bien. |
| C15. Me preocupa no poder terminar el curso/me preocupa que los estudiantes tengan dificultades para terminar el curso. |

**ESCALA DE AFRONTAMIENTO**

| ***Apoyo/Consecuencias*** |
| --- |
| Af2. Cuando en estos días me siento emocionalmente triste o desanimado he buscado apoyo social en mis amigos/compañeros/ familiares. |
| Af7. Pienso que esta situación por la que estamos pasando me hará más fuerte y me ayudará a valorar lo que realmente merece la pena. |
| Af10. Todos los días dedico un tiempo a hablar con algún amigo, familiar por teléfono. |
| C1. Me siento emocionalmente sobrecargado con mis estudios/trabajo. |
| C2. Al final del día me siento más cansado que en situaciones normales. |
| C8. La docencia online que he tenido que asumir de manera forzada me genera más estrés que la docencia presencial. |

P (Preocupación, tensión y estrés); Af (Estrategias de afrontamiento; C (Cambios en la vida)
